# Event and Comment

**Published:** 1912-03-15

**Authors:** 


### DENTAL REGISTER.

Vol. LXVI. March 15, 1912. No. 3

### EVENT AND COMMENT.

EDITOR BETHEL. Dr. L. P. Bethel the editor of the widely read *Dental Summary* is celebrating in the February issue of that journal his twenty five years service as an editor. He tells us of some of the trials and troubles of an editor, but not all, and mentions some of the history made during this period. He does not tell us of the joys of an editorial life, and we sometimes wonder if there be such. There must be something in the life that will keep a sensible man grinding out copy with a ceaseless regularity that must tend to insanity if there were no high and inspiring motives. It can not be the munificence of the monetary compensation, for very obvious reasons; and we are loath to think it can be the satisfaction of an inordinate egotism to keep oneself always in the public eye. We are inclined rather to believe it must be the excitement, such as keeps the locomotive engineer or the aviator at his post; as from his position of duty and opportunity he is sustained and strengthened by the knowledge that he has a breadth of view and experience which is not given to all men. And out of the benevolent disposition automatically generated by his constant efforts to make visible to others what he sees, that fills him with a constantly increasing desire to multiply his efforts to make his life still more useful to his professional confreres. We are sure there must be some such delight or joy to sustain ones spirit and keep him faithful, and no other motive appeals to us as so deserving of the long and faithful service which Dr. Bethel has given to his task. We extend our congratulations and appreciation of his generous work and trust he may live to celebrate his golden anniversary.

INSTITUTE OF DENTAL PEDAGOGICS. The nineteenth annual meeting of this society was held January 24th, 1912, at Chicago. The two days previous were given over to a clinic and meeting of the Chicago Dental Society, consequently there was an exceptionally large gathering of dental teachers from all parts of the country. The papers and discussions were exceptionally good and the technical exhibits from the various schools were more like the former times when the technical exhibits were the predominating feature of the meeting. At this meeting Dr. Wm. E. Bebb, of Los Angeles, California, exhibited his collection of animal crania, said to be the finest private collection in the country. An important and interesting featuie of the meeting was a visit to the dental colleges, all of which arranged their work so that practically every feature could be seen by the visitors. This proved to be a very valuable and instructive part of the programme. A proposition was started to change the name to The American Association of Dental Teachers. Just what necessity there may be for this change is not very clearly set forth. Is it contemplated that the character of work done be simplified or changed. Is it contemplated that the Association go into professional politics or fraternal insurance? Pedagogy is a good name for teachers meetings, or it should be.

THE TAGGART SUIT. It is stated that Dr. Taggart got a favorable decision on his patent suit at the rehearing, but we are unable to give the particulars. If the decision stands it will now be in order for everyone to comply with Dr. Taggart's terms and take out a license or quit using the casting process. We understand the defendants have appealed to the higher court.

